# Comparison of Nasal and Bronchial Epithelial Cells Obtained from Patients with COPD

**DOI:** 10.1371/journal.pone.0032924

**Published:** 2012-03-06

**Authors:** David M. Comer, J. Stuart Elborn, Madeleine Ennis

**Affiliations:** 1 Centre for Infection and Immunity, School of Medicine, Dentistry and Biomedical Sciences, The Queen's University of Belfast, Belfast, United Kingdom; 2 Respiratory Department, Belfast City Hospital, Belfast, United Kingdom; University of Giessen Lung Center, Germany

## Abstract

For in vitro studies of airway pathophysiology, primary epithelial cells have many advantages over immortalised cell lines. Nasal epithelial cells are easier to obtain than bronchial epithelial cells and can be used as an alternative for in vitro studies. Our objective was to compare nasal and bronchial epithelial cells from subjects with COPD to establish if these cells respond similarly to pro-inflammatory stimuli. Cell cultures from paired nasal and bronchial brushings (21 subjects) were incubated with cigarette smoke extract (CSE) prior to stimulation with *Pseudomonas aeruginosa* lipopolysaccharide. IL-6 and IL-8 were measured by ELISA and Toll-like receptor 4 (TLR-4) message and expression by RT-PCR and FACS respectively. IL-8 release correlated significantly between the two cell types. IL-6 secretion was significantly less from bronchial compared to nasal epithelial cells and secreted concentrations did not correlate. A 4 h CSE incubation was immunosuppressive for both nasal and bronchial cells, however prolonged incubation for 24 h was pro-inflammatory solely for the nasal cells. CSE reduced TLR-4 expression in bronchial cells only after 24 h, and was without effect on mRNA expression. In subjects with COPD, nasal epithelial cells cannot substitute for in vitro bronchial epithelial cells in airway inflammation studies.

## Introduction

The airway epithelium is a vital part of our immune defences against pathogens, allergens and other noxious agents such as cigarette smoke. Airway epithelial cells have the ability to synthesise and secrete cytokines, chemokines, and anti-microbial substances. Profound differences in the constitutive and stimulated expression and secretion of IL-8 by airway epithelial cells from healthy volunteers and patients with chronic obstructive pulmonary disease (COPD) have been described [1].

There is an increasing body of research dedicated to the study of primary epithelial cells from patients with asthma [2] and cystic fibrosis [3], with fewer studies examining epithelial cells from patients with COPD [1], [4]. Airway epithelial cells have usually been obtained from transplant lungs or surgical specimens, but the use of nasal or bronchial brushings has now become increasingly popular [3], [5], [6]. Nasal brushings are less invasive, more acceptable for patients and easier to perform than bronchial brushings. However, few studies have directly compared data from nasal and bronchial brushings. McDougall *–et al.* reported that nasal cells could be used as surrogates for bronchial cells in studies of airway inflammation [6]. Furthermore, MacRedmond *–et al.* demonstrated a strong correlation in TLR-4 mRNA expression from cells obtained from the upper and lower respiratory tract, both obtained by brush sampling, in a group of COPD patients [5]. In contrast, Thavagnanam and co-workers found differences between nasal and bronchial epithelial cells from subjects with and without asthma under basal conditions and after IL-13 treatment [7].

There is increasing evidence that the disease process in COPD is not confined to the lower airways. Sinonasal symptoms in COPD have been reported ranging from 75% [8] to as much as 88% [9]. These findings highlight that the pathophysiology of COPD is not merely confined to the lower airways. This is not particularly surprising, as although smokers may vary in terms of the frequency and depth of inhalation of cigarette smoke, for many of these individuals the entire airway is exposed to volatile cigarette smoke.

To investigate the action of cigarette smoke extract (CSE) on epithelial cells, we compared the responses of paired nasal and bronchial epithelial cells obtained from patients with COPD. We hypothesised that nasal and bronchial epithelial cells, obtained by brushings at each site, demonstrate similar release of IL-8 and IL-6 after LPS stimulation.

## Materials and Methods

### Study Subjects

Patients with a diagnosis of mild COPD according to the British Thoracic Society guidelines were included [10]. All subjects required an elective bronchoscopy as part of their clinical investigation. Subjects with bronchiectasis, a clinical diagnosis of lung carcinoma, or other significant pulmonary or nasal pathology were excluded. This study was approved by the Office for Research Ethics Committees Northern Ireland and all participants provided written informed consent (REC: 09/NIR03/42).

### Nasal and bronchial brushings

Bilateral nasal brushings were performed using a bronchial cytology brush (TeleMed Systems Inc., MA, USA) from the medial aspect of the inferior turbinate as previously described [11]. During bronchoscopy, four bronchial brushings in the third generation bronchus were obtained. Nasal and bronchial cells were then expanded in bronchial epithelial growth medium (BEGM, Promocell, Germany) [11].

Cells (passage 2–3; 6×10^4^ cells per well) were seeded in purified bovine collagen coated (PureCol; Advanced Biomatrix) 12–well plates and cultured for 24 h. Cells were stimulated with LPS from *Pseudomonas aeruginosa* (Sigma-Aldrich) either with or without CSE pretreatment for 4 h or 24 h as outlined in the results section. Separate cultures were treated with acrolein (up to 30 µM for 4 h). Media were collected for the determination of cytokine production, and cells were used for mRNA or protein expression studies. After appropriate stimulation, cell–free supernatants were collected and stored at −20° for future IL-8 and IL-6 measurement using the Duoset ELISA kits (R&D Systems) according to the manufacturer's instructions.

For experiments in air-liquid interface (ALI) cultures, cells were seeded onto collagen coated Transwells (Corning Inc, USA) at a seeding density of 1.5×10^5^ cells per well and grown until cultures became confluent and developed tight junctions. At this stage the apical media was removed and the cell cultures fed basolaterally only on alternate days for 28 days. Mucus secretion was apparent after approximately 14 days in culture.

### Immunofluorescence

Cells were seeded on coverslips at a density of 1×10^5^ cells/ml. The following day, cells were fixed in 4% PFA and washed in PBS. After permeabilisation with 0.2% Triton-X 100 (Sigma, Dorset, UK), cells were treated with a 1∶100 dilution of rabbit anti-cytokeratin-5 primary antibody (Abcam, Cambridge, UK) overnight at 4°C. The primary antibody was detected using a 1∶500 dilution of Alexafluor 568 goat anti-rabbit IgG (Invitrogen Ltd., Paisley, UK). Nuclei were stained with DAPI (Vector Laboratories, Peterborough, UK). Images were captured using LAS AF (Leica) acquisition software.

### Cigarette smoke extract preparation

CSE was prepared by a modification of the method of Richter *et al.* [12] Briefly, one Marlboro cigarette (0.8 mg nicotine; 10 mg Tar; 10 mg carbon monoxide) was combusted with a modified syringe-driven apparatus. The smoke was bubbled through 25 ml of media over 5 minutes by drawing 35-ml volume of smoke every 15 s. The resulting suspension was filtered through a 0.2 µm pore-size filter to remove large particles and bacteria. This solution was regarded as “100% CSE” and was freshly generated for each experiment, and subsequently diluted with culture medium to obtain a final 5% working concentration. CSE was used immediately to avoid break-down or evaporation of components. The optical density of CSE measured at 450 nm did not vary significantly when comparing a series of 5% CSE preparations (OD approx. 0.25).

### FACS analysis

Cell surface expression of TLR-4 was determined by FACS analysis. In brief, cells were detached from wells by incubating with cell dissociation fluid (Sigma-Aldrich) in PBS at 37°C. For the analysis of intracellular TLR-4, cells were permeabilised and fixed by incubating them with fixation and permeabilization buffer (eBioscience, USA) as per supplier's instructions and then washed twice with staining solution containing 1.0% BSA and 0.02% sodium azide in PBS. Cells, adjusted to 1×10^5^ cells per 100 µl in PBS/1% BSA, were subsequently stained in darkness for 30 min at 4°C with phycoerthrin (PE)-conjugated anti-TLR-4 monoclonal antibodies or PE-mouse isotype control (eBioscience, USA). Analysis of 10,000 events was performed using an Epics XL flow cytometer (Beckman Coulter, UK Ltd). Cells were initially gated on the basis of forward and side scatter characteristics. Results are expressed as relative mean fluorescence intensity (rMFI  =  monoclonal antibody/corresponding isotype control) as no bimodal distribution was found.

### RT- PCR Analysis of TLR-4 mRNA

RNA was isolated using the RNeasy Mini kit (Qiagen, UK). RNA quality and quantity were evaluated by UV spectrophotometry. cDNA was prepared by first-strand cDNA synthesis from 1 µg total RNA using Roche first strand cDNA synthesis kit for RT-PCR according to manufacturer's instructions (First strand cDNA synthesis Kit, Roche). For PCR quantification, 1 µl of cDNA reaction were amplified in a 10 µl standard PCR in a thermo-Fast 96-well detection plate and run on the spectrofluorometric thermal cycler AB 1200. PCR for GAPDH and TLR-4 were performed in parallel. The threshold cycle number (Ct) for each sample was determined. The gene-specific Ct for each sample was corrected by subtracting the Ct for the house-keeping GAPDH (ΔCt). Untreated samples were chosen as the reference samples and their ΔCt values were subtracted to the ΔCt from the treated samples to obtain the ΔΔCt values. Finally, sample mRNA abundance relative to control mRNA abundance was calculated by the formula 2^−(ΔΔCt)^.

### Statistics

Statistical analysis was performed using SPSS version 17.0 (SPSS inc., Chicago, IL, USA). Data are presented as median values ± interquartile range. Comparisons between groups were performed using the nonparametric Kruskal-Wallis test for multiple comparisons and the Mann-Whitney test for two groups. A *p* value of less than 0.05 was considered significant.

## Results

### Patient demographics

21 patients (12 male, 9 female) with a mean age of 68 years were included in the study. The majority were referred after an episode of haemoptysis for exclusion of an underlying malignancy. The mean FEV_1_ was 1.36 L (54% predicted), FVC 2.69 L and FEV_1_/FVC ratio 50.5. None of the patients had taken oral corticosteroids for at least 8 weeks prior to the study, and no individual had bronchodilator reversibility of greater than 10%.

### Cell culture

**Figure 1:**
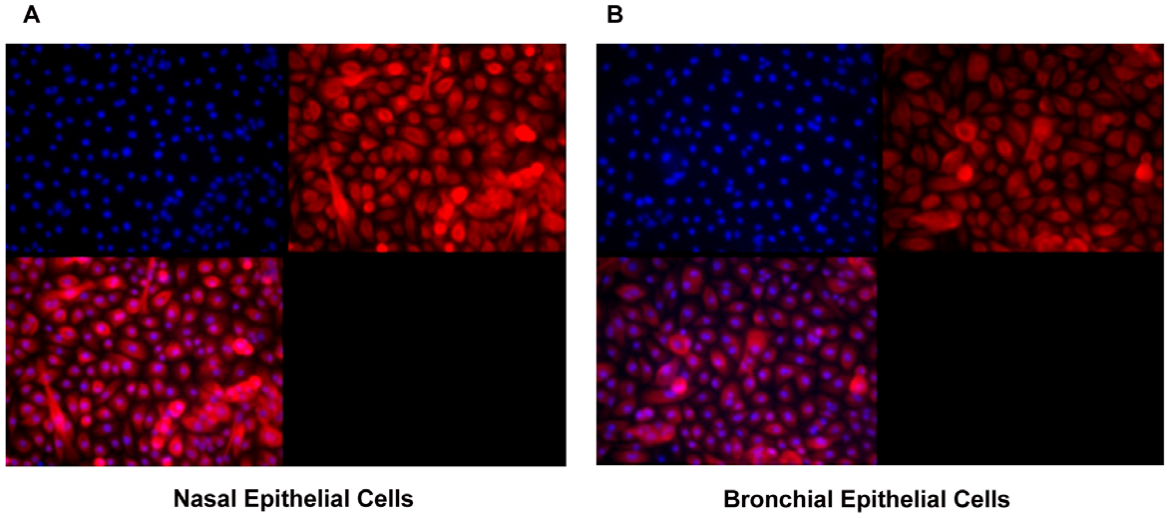
Immunocytochemistry for cytokeratin 5 in nasal and bronchial epithelial cell cultures. Primary nasal (A) and bronchial (B) epithelial cell cultures were stained with a rabbit anti-human antibody against cytokeratin 5 (1∶100). The primary antibody was detected using a secondary antibody coupled to Alexafluor 568 (1∶500). Nuclei were stained blue with DAPI (×40).

Bronchial and nasal epithelial cells obtained by brushings from each site reached confluence after 10–14 days, with a trend towards a longer time required for the bronchial epithelial cells. Cell cultures selected at random confirmed epithelial phenotype of the cells (Figure 1). The success rate was 80–90% from obtaining cells to growing to sufficient numbers to facilitate experiments.

### Mediator release

**Figure 2:**
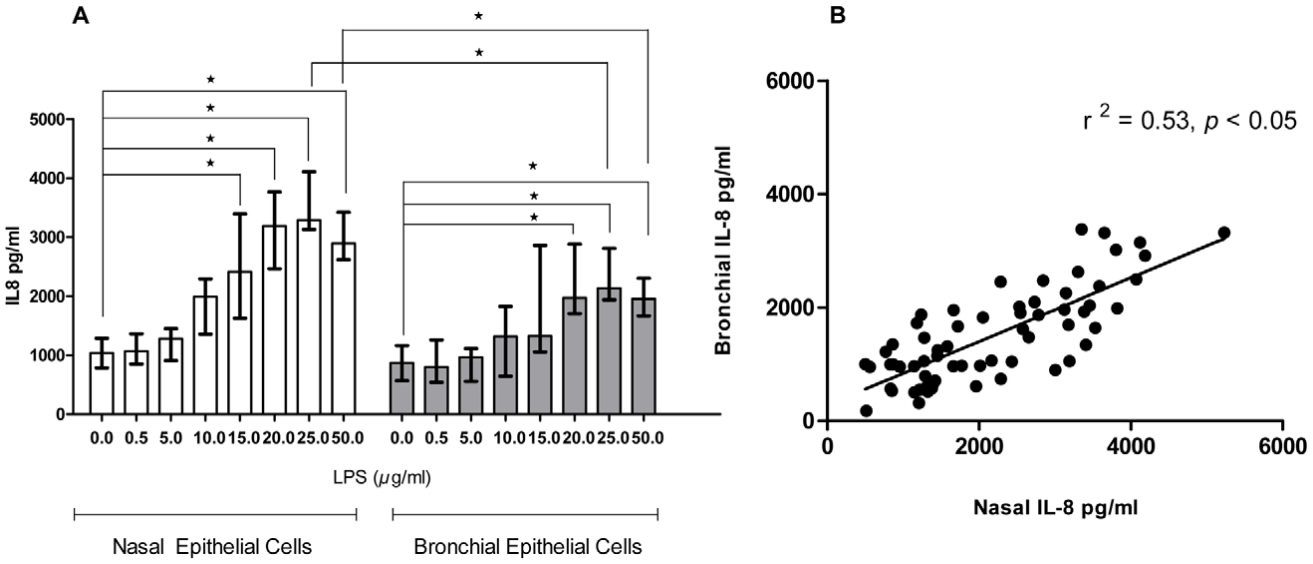
(A) IL-8 dose response and (B) linear regression analysis from paired nasal and bronchial epithelial cells from COPD subjects after 24 h treatment with LPS. Paired nasal and bronchial epithelial cells were with various concentrations of LPS [5–25 µg/ml] for 24 h (n = 8). Supernatants were collected and assessed for IL-8 by ELISA in all cases. Data are displayed as median (interquartile range). * indicates a significant difference (*p*<0.05).

**Figure 3:**
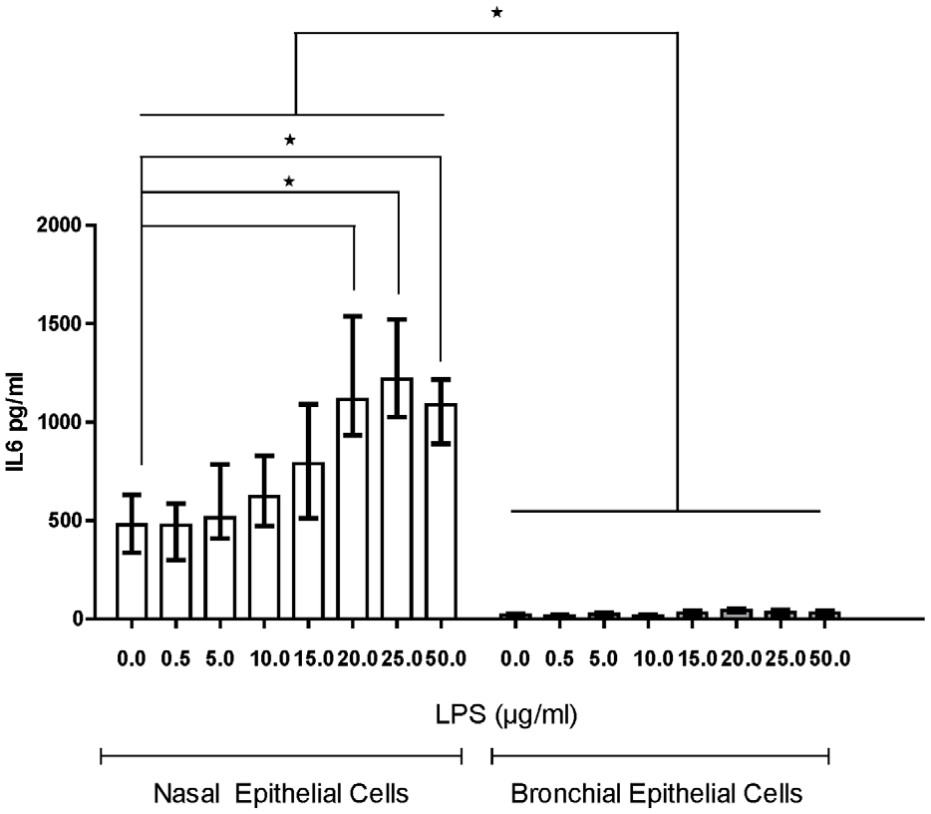
IL-6 dose response from paired PNECs and PBECs from COPD subjects after 24 h treatment with LPS. Paired nasal and bronchial epithelial cells were with various concentrations of LPS [0–25 µg/ml] for 24 h (n = 8). Supernatants were collected and assessed for IL-6 by ELISA in all cases. Data are displayed as median (interquartile range). * indicates a significant difference (*p*<0.05).

Stimulation with *P aeruginosa* LPS (5–25 µg/ml) elicited a dose dependent release of IL-8 from both nasal and bronchial epithelial cells (Figure 2A). Although the amount of IL-8 secreted by the nasal cells was higher than that secreted by the bronchial cells, the release was correlated (Figure 2B). IL-6 secretion was less than that of IL-8, with minimal secretion from the bronchial epithelial cultures (Figure 3).

**Figure 4:**
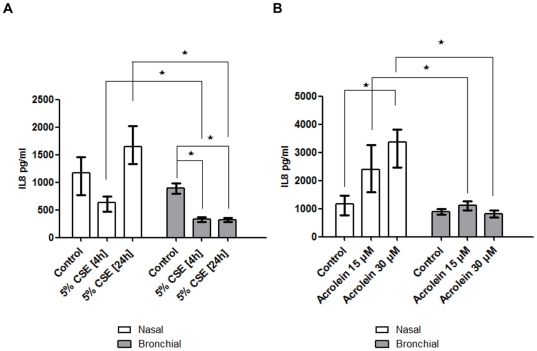
Effects of (A) 5% CSE and (B) acrolein on IL-8 release from nasal and bronchial epithelial cultures. Cells were treated 5% CSE prepared with a single cigarette for 4 h or 24 h (n = 5). In separate experiments, cells were treated with increasing concentrations of acrolein for 4 h. Supernatants were collected and assessed for IL-8 by ELISA in all cases. Data are displayed as median (interquartile range). * indicates a significant difference (*p*<0.05).

Preliminary experiments indicated that the 5% CSE chosen for the studies did not cause cell death. Higher concentrations than 5% CSE caused unacceptable cytotoxicity for both bronchial and nasal epithelial cells (Figure S1A). A 4 h preincubation with CSE inhibited basal IL-8 release from both nasal and bronchial cells (only reaching statistical significance for the bronchial epithelial cells). However, there was a trend for increased release after a 24 h preincubation with CSE (5%) for the nasal cells only, inhibiting release from the bronchial cells (Figure 4A). Incubation with acrolein for 4 h also gave divergent results, stimulating IL-8 release from the nasal cells, but having no effect on IL-8 release from bronchial cells (Figure 4B).

After treating primary bronchial epithelial cells with 25 µg/ml LPS for 24 h in the ALI culture model there was a 1.29 fold increase in IL-8 release. This was reduced to 0.53 fold change relative to control after pre-treatment with CSE for 24 h. The corresponding values for the submerged model were 2.72 and 1.84 respectively. Control release of IL-8 was 33,449 pg/ml and 893 pg/ml in the ALI and submerged models respectively.

### Protein expression of TLR-4 is reduced in bronchial epithelial cell cultures after treatment with CSE

**Figure 5:**
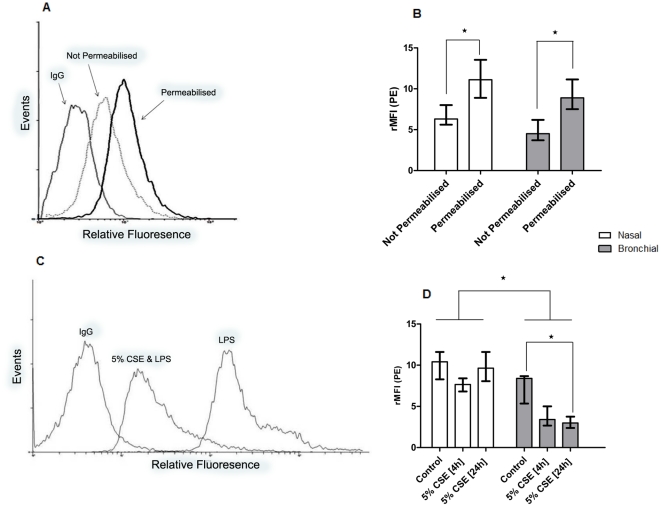
Representative FACS histograms demonstrating intracellular-location of TLR-4, and a reduced total TLR-4 after CSE treatment. Localisation of Toll-like receptor 4 (TLR-4). (A): Representative histogram for surface and cytoplasmic staining for IgG and TLR-4 in bronchial epithelial cells. (B): rMFI for TLR-4 in permeabilised and non-permeabilised nasal and bronchial epithelial cells. (C): Staining for TLR-4 in permeabilised bronchial epithelial cells after stimulated with 25 µg/ml *P aeruginosa* LPS, with or without 24 h pre-treatment with CSE. (D): rMFI for TLR-4 was lower in bronchial epithelial cells than for nasal epithelial cells (for control, 4 h and 24 h CSE treatment). A 24 h exposure to CSE significantly decreased rMFI for bronchial, but not for nasal, epithelial cells. Data are displayed as median (interquartile range). * indicates a significant difference (*p*<0.05).

TLR-4 was predominantly expressed intracellularly in both nasal (Figure S1B) and bronchial epithelial cells (Figure 5A & 5B), and therefore cells were permeabilised for all measurements after stimulation. In bronchial epithelial cell cultures, the rMFI for TLR-4 was significantly decreased after treatment with CSE for 24 h (Figure 5C), but not after 4 h (Figure S1C). There were no significant changes in the nasal epithelial cell cultures for either exposure time (Figure 5D).

### mRNA expression of TLR-4

**Figure 6:**
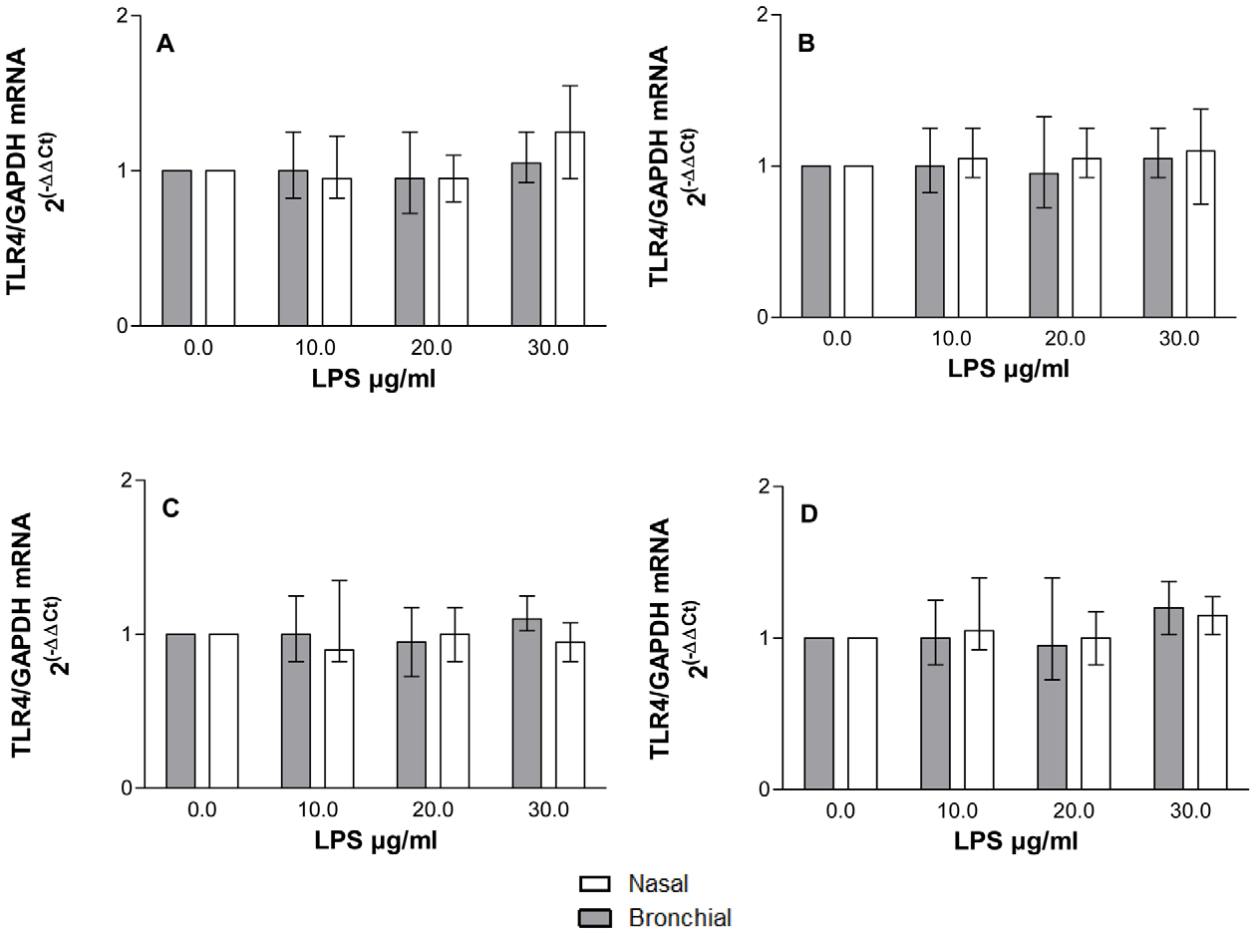
Effect of LPS ±5% CSE pre-treatment on TLR-4 mRNA expression. Nasal epithelial cell cultures were pretreated with 5% CSE prepared for (A) 4 h,(B) 24 h or vehicle, and subsequently stimulated with *P aeruginosa* LPS [10–30 µg/ml]for 24 h. Experiments were repeated in bronchial epithelial cell cultures, again for (C) 4 h and (D) 24 h 5% CSE exposure.

Our results show similar mRNA expression of TLR-4 after treatment with *P aeruginosa* LPS, either with or without pre-treatment with CSE for 4 h or 24 h (Figure 6).

## Discussion

These experiments tested the hypothesis that the inflammatory responses are related in the upper and lower airways. The responses were monitored by measuring IL-6 and IL-8 release from cell cultures, under resting and stimulated conditions, as well as examining TLR-4 message and expression.

The principle of a united airway proposes that clinically the upper and lower airway mirror each other in terms of their individual disease manifestations [13]. The use of nasal epithelial cells in cell culture models as an alternative to bronchial epithelial cells is dependent on a correlation between their respective inflammatory responses. In order to address this hypothesis, McDougall *et al.*, demonstrated that although there was a significant difference in absolute mediator levels in paired nasal and bronchial monolayer cell cultures, there was a correlation in the inflammatory cytokine levels (both basally and after stimulation) [6]. However, their 35 subjects were heterogeneous both in terms of the age range and the spectrum of underlying lung pathology. In contrast, a satisfactory correlation was not present for basal and stimulated mediator release for bronchial and nasal epithelial cells from asthmatic and non-asthmatic children [7].

Our data extends previous findings using cystic fibrosis tracheobronchial epithelial cells indicating that IL-8 secretion was greater than IL-6 secretion (basally and after stimulation) [14]. Although the magnitude of IL-8 release was different in both bronchial and nasal cell cultures (Figure 2A), the release was strongly correlated (Figure 2B). In contrast, although there was a significant IL-6 dose dependent release from the nasal epithelial cells after stimulation with *P aeruginosa* LPS, this was not evident in the bronchial cell cultures (Figure 3). The responses of nasal and bronchial epithelial cell cultures to treatment with acrolein, an important constituent of cigarette smoke, were also divergent. Although the nasal cultures were stimulated by acrolein after a 4 h incubation period, there was no effect on the bronchial cell cultures (Figure 4B).

Data on the effects of acrolein in various cell culture models are inconsistent. Treating nasal epithelial cell cultures (from subjects with chronic rhinosinusitis) with acrolein suppresses basal IL-8 release [15]. Acrolein has also been reported to stimulate IL-8 release from cultured small airway epithelial cells [16]. Other groups have indicated that acrolein inhibits IL-8 release in primary bronchial epithelial cells, and in immortalised bronchial epithelial cell lines [17]. Similar concentrations of acrolein were used in all of these studies (5–30 µM range). Thus, although acrolein has profound effects on epithelial cells, these responses are critically dependent on the cell type. Overall, these findings emphasise the importance to study cells from the relevant anatomical location for any given disease.

The higher IL-8 release from nasal cultures is not particularly surprising. Nasal epithelial cells have a greater exposure to respirable inflammatory particles in the environment. Thus, the upper airway acts to protect the more sensitive lower airways from infectious agents, allergens and pollutants in the environment. Devalia *el al*. provided further evidence to unite the nasal and bronchial epithelium by comparing bronchial and nasal epithelial cells acquired from tissue from subjects attending for turbinectomy and lung surgery respectively, and reported few differences between the nasal and bronchial cells with respect to their morphology or ciliary activity [18]. However, the samples were not obtained from the same individuals. From the published data, in adults, it appears that nasal epithelial cells can be used as a satisfactory surrogate for bronchial epithelial cells for many morphological and functional endpoints. However, we have shown this is not valid for all situations.

Although we have shown differences in soluble mediator release from epithelial cells, previous data suggest that there is a conserved response to cigarette smoke exposure at both sites in terms of smoking-related gene expression, in particular for those genes related to oxidative stress and wound healing [19], [20]. However, in a subset of genes, expression was affected more dramatically in the bronchial than in the nasal epithelium [20]. We found that exposure to CSE reduced intracellular TLR-4 (Figure 5C), without modifying mRNA expression (Figure 6).

The localisation of TLR-4 is controversial. There are a number of studies which indicate that this receptor is present at the cell surface and that it is functional at this site, whereas other studies suggest that it has an exclusive intracellular localisation and that the activation process of TLR-4 requires internalisation of LPS. These differences may be explained by the cell type used. A predominant surface expression was reported in studies using CF airway cell lines and primary alveolar type 2 cells [21], [22], whereas intracellular expression was found with human bronchial epithelial cell lines and the A549 alveolar carcinoma epithelial cell line [23]. Our FACS data indicates that in COPD bronchial epithelial cells, TLR-4 is present both on the surface and intracellularly, although predominantly in the latter location. Interestingly, a reduced inflammatory response to stimulation with LPS from Gram-negative bacteria in cystic fibrosis airway epithelial cells has previously been attributed to a lower surface expression of surface TLR-4, a finding which is reflective of our experiments using CSE [24]. This reduced surface expression of TLR may in fact be an appropriate response, serving to mitigate damaging inflammation in the lower airways.

The reported effects of CSE on TLR-4 expression are equally conflicting. A dose dependent down-regulation of TLR-4 in A549 cells, at both mRNA and protein expression, after stimulation with CSE has been reported [5]. In contrast, a later publication suggested that CSE increases TLR-4 expression in BEAS-2B cells [25]. Similar CSE concentrations were used, but the exposure time differed with the 4 h exposure causing down-regulation, and the 18 h causing up-regulation of TLR-4 expression.

All of our experiments are performed on cells at passage 2–3. These cell cultures require approximately two weeks to become confluent in T75 flasks during which time the media is changed on alternate days. The environment both within the nose and the main airways is therefore unlikely to have any influence on the phenotype of the cell cultures themselves. The higher soluble mediator release from the nasal cultures compared to the bronchial cultures can therefore be attributed to intrinsic phenotype, rather than attributed to their site of retrieval.

Our results demonstrate that a brief CSE exposure suppresses the inflammatory response in COPD nasal epithelial cells to stimulation with LPS, and that a more prolonged CSE exposure for 24 h heightens the inflammatory response, although neither response reached statistical significance (Figure 4A). These *in vitro* findings suggesting a pro-inflammatory effect of prolonged CSE exposure in this cell type is reflective of *in-vivo* research whereby mice exposed to volatile smoke for 3 days developed a marked inflammatory cell recruitment in the airways which interestingly was TLR4 dependent [26]. In contrast, we have demonstrated that treating primary human bronchial epithelial cells with CSE in the same experimental conditions was solely immunosuppressive. These findings are also similar to mice exposed to mainstream cigarette smoke where a different inflammatory response in the upper and lower airways was apparent [27]. We adopted 2 exposure times in order to facilitate a comparison of our data with other published work. The concentrations of CSE used can range from 100% CSE for 15 minutes [28] to a 1% CSE for 24 hours (in those studies which use a single cigarette to prepare the initial “100%” stock CSE) [29]. A study exposing primary human nasal epithelial cells to CSE for 1 h, 2 h, and 4 h reported a time and dose-dependent cytotoxicity of CSE [30].

The methods used in CSE research are strikingly diverse which may account for the conflicting reports whether CSE has pro-inflammatory effects [28], [31]. Different cigarettes ranging from the international reference cigarette KY1R3F [32] to popular brands of cigarettes are used [33]. In some cases, the particular type of cigarette has not been defined [34]. In one study, the cigarettes used did not have a filter [35], whereas in others the filter was manually removed [12], [32]. The presence of a filter actually increases the production of tar phase and stable free radicals that are present in mainstream cigarette smoke [36]. In the majority of studies, cigarette smoke is bubbled through tissue culture medium without serum [33], [37], or alternatively phosphate buffered saline was used as the diluent [32]. The lack of consensus in terms of the inherent capacity for CSE to induce a pro-inflammatory response in epithelial cells may be attributable to an absence of broadly accepted standardised protocol for the collection and processing of cigarette smoke.

Submerged cell culture models are most widely used in CSE research [28], [29], [38], [39], [40], [41]. However, ALI cultures have become increasingly popular particular when morphological endpoints are evaluated [42], These cell cultures form many layers, and after 2 weeks in culture develop beating cilia and goblet cells. We have successfully cultured bronchial epithelial cells in this model (in conjunction with submerged cultures) and proceeded to compare responses after stimulation with 25 µg/ml LPS, with or without CSE pre-treatment for 24 h. Although both cultures were stimulated with LPS, this was more pronounced in the submerged model. A limitation of the technique we use to obtain primary bronchial epithelial cells is the relatively low yield of cells obtained in comparison to those groups who obtain cells from transplanted lungs [14]. This limits the number of experiments that we can perform.

In conclusion, our data indicate that although nasal epithelial cells can be used as suitable surrogates for bronchial epithelial cells for IL-8 responses to *P aeruginosa* LPS, but not for IL-6 release, responses to CSE, acrolein, or TLR-4 expression. The combination of a relatively non-invasive and inexpensive nasal brushing procedure facilitates a unique opportunity to study the specific patterns of inflammatory responses in a number of settings, but nasal epithelial cell responses cannot be extrapolated to those of the lower airway in subjects with COPD when these particular endpoints are considered. Investigating cytokine protein panels or microarray analysis would serve to extend these findings in a broader context.

## Supporting Information

Figure S1 **Localisation of Toll-like receptor 4 (TLR-4).** (A) CSE dose dependent cytotoxicity for nasal and bronchial epithelial cells determined using the 3-(4,5-dimethylthiazol-2-yl)-2,5-diphenyl tetrazolium bromide (MTT) assay. (B) Representative histogram for surface and cytoplasmic staining for IgG and TLR-4 in nasal epithelial cells. (C) TLR-4 expression in PBECs after 4 h and 24 h CSE exposure, both prior to LPS stimulation.
(TIFF)
