# Supplementary figures and images for: Comparison of Nasal and Bronchial Epithelial Cells Obtained from Patients with COPD
Source: PLoS One. 2012 Mar 6;7(3):e32924. doi: 10.1371/journal.pone.0032924 (PMC3295776; doi:10.1371/journal.pone.0032924)

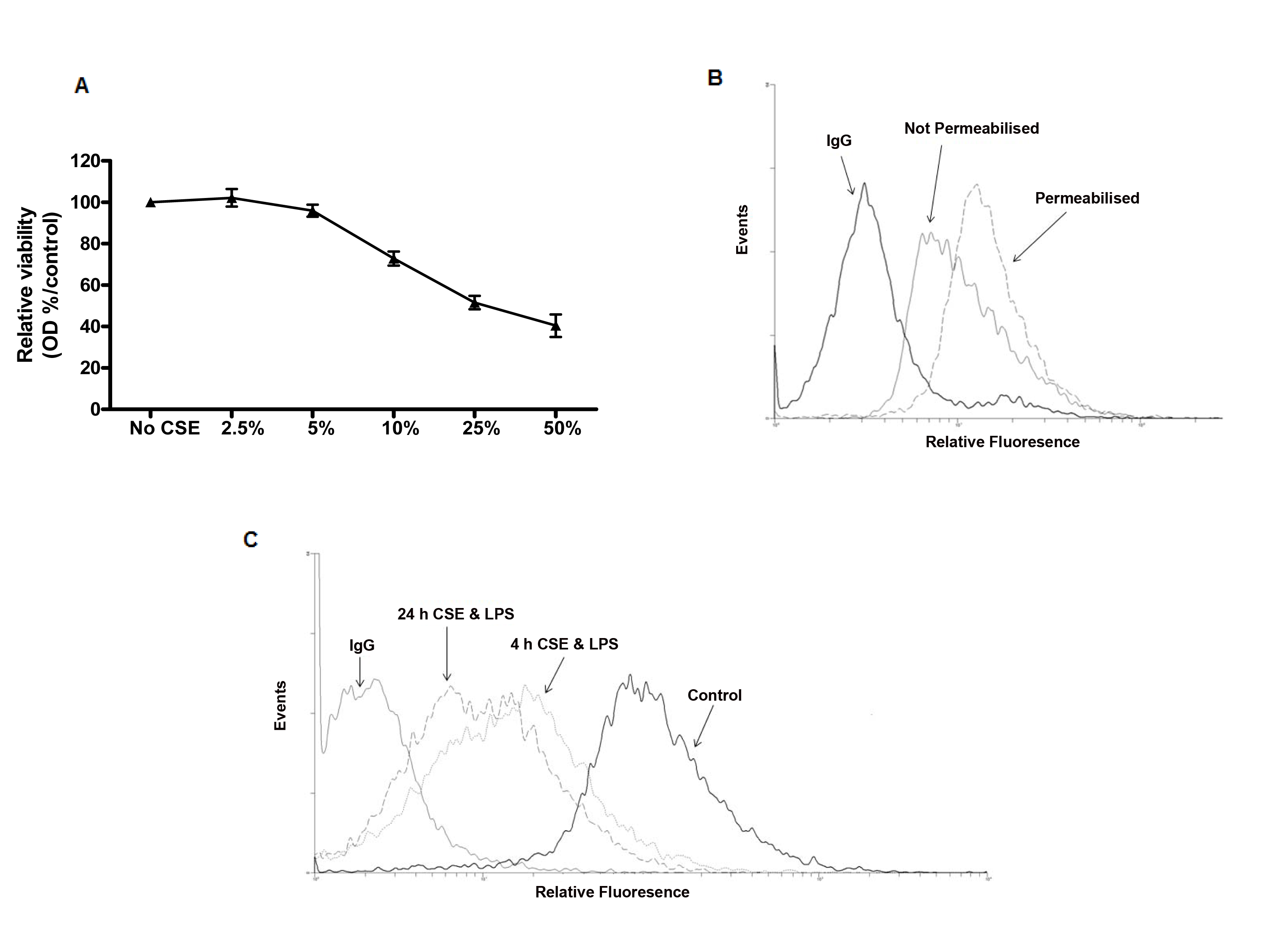

Supplement: Figure S1 — Localisation of Toll-like receptor 4 (TLR-4). (A) CSE dose dependent cytotoxicity for nasal and bronchial epithelial cells determined using the 3-(4,5-dimethylthiazol-2-yl)-2,5-diphenyl tetrazolium bromide (MTT) assay. (B) Representative histogram for surface and cytoplasmic staining for IgG and TLR-4 in nasal epithelial cells. (C) TLR-4 expression in PBECs after 4 h and 24 h CSE exposure, both prior to LPS stimulation. (TIFF) [file pone.0032924.s001.tiff]
